# Genomic landscape of a mouse model of diffuse-type gastric adenocarcinoma

**DOI:** 10.1007/s10120-021-01226-0

**Published:** 2021-08-13

**Authors:** Menghua Zhang, Itsuki Sugita, Daisuke Komura, Hiroto Katoh, Shu Shimada, Johji Inazawa, Shinji Tanaka, Shumpei Ishikawa

**Affiliations:** 1 Department of Preventive Medicine, Graduate School of Medicine, The University of Tokyo, 7-3-1 Hongo, Bunkyo-ku, Tokyo, 1130033 Japan; 2 Department of Molecular Cytogenetics, Medical Research Institute, Tokyo Medical and Dental University, Tokyo, Japan; 3 Department of Molecular Oncology, Graduate School of Medicine, Tokyo Medical and Dental University, Tokyo, Japan

**Keywords:** Gastric cancer, Mouse model, Whole-genome sequence

## Abstract

**Background:**

There is a need for a model of diffuse-type gastric cancer that captures the features of the disease, facilitates the study of its mechanisms, and aids the development of potential therapies. One such model may be *Cdh1* and *Trp53* double conditional knockout (DCKO) mice, which have histopathological features similar to those of human diffuse-type gastric cancer. However, a genomic profile of this mouse model has yet to be completed.

**Methods:**

Whole-genome sequences of tumors from eight DCKO mice were analyzed and their molecular features were compared with those of human gastric adenocarcinoma.

**Results:**

DCKO mice gastric cancers harbored single nucleotide variations and indel patterns comparable to those of human genomically stable gastric cancers, whereas their copy number variation fraction and ploidy were more similar to human chromosomal instability gastric cancers (perhaps due to *Trp53* knockout). Copy number variations dominated changes in cancer-related genes in DCKO mice, with typical high-level amplifications observed for oncogenic drivers, e.g., *Myc*, *Ccnd1*, and *Cdks*, as well as gastrointestinal transcription factors, e.g., *Gata4*, *Foxa1,* and *Sox9*. Interestingly, frequent alterations in gastrointestinal transcription factors in DCKO mice indicated their potential role in tumorigenesis. Furthermore, mouse gastric cancer had a reproducible but smaller number of mutational signatures than human gastric cancer, including the potentially acid-related signature 17, indicating shared tumorigenic etiologies in humans and mice.

**Conclusions:**

*Cdh1/Trp53* DCKO mice have similar genomic features to those found in human gastric cancer; hence, this is a suitable model for further studies of diffuse-type gastric cancer mechanisms and therapies.

**Supplementary Information:**

The online version contains supplementary material available at 10.1007/s10120-021-01226-0.

## Introduction

Gastric cancer (GC) is the fifth most prevalent cancer and ranks third in cancer deaths globally; incidence varies among regions, but is highest in eastern Asia [1, 2]. Traditionally, GCs have been classified into intestinal and diffuse types based on their histological characteristics, and diffuse-type GC (DGC) is associated with poorer prognosis than intestinal-type GC. Recently, the cancer genome atlas (TCGA) defined four molecular subtypes: genomically stable (GS), chromosomal instability (CIN), Epstein–Barr virus (EBV), and microsatellite instable (MSI). Among these, GS is dominated by DGC [3]. Conversely, DGC is not GS-dominant; only approximately 43% of DGC cases were classified as GS, approximately 40% showed CIN, and the remaining were classified as EBV or MSI. The incidence of DGC varies among regions and ethnicities, which accounts for approximately 20–40% of GCs [4, 5]. However, current GC treatment does not depend on such classifications, and chemotherapy remains the main first option besides resection, whereas novel targeting therapy and immunotherapy benefit a limited number of patients [6]. Indeed, due to the heterogeneity of GC, current treatments are unsatisfactory. Moreover, the precise mechanism underlying DGC development is unknown; typically, no alteration features suitable for existent molecular targeting therapies or immunotherapy have been observed. Therefore, a DGC model is required to facilitate research into disease mechanisms and treatments.

Mouse cancer models are essential to cancer research. In particular, genetically engineered mouse models that successfully capture human cancer complexities provide opportunities for drug target validation in natural microenvironments and are the only preclinical platform for immunomodulatory therapy studies [7]. In *Atp4b*-Cre^+^; *Cdh1*^*loxP/loxP*^*; Trp53*^*loxP/loxP*^ double conditional knockout (DCKO) mice, a driver-gene knockout DGC model [8], *Cdh1* and *Trp53* (mouse orthologs of *CDH1* and *TP53*) are specifically knocked out in mouse parietal cells. *CDH1*, encoding the cell adherence molecule E-cadherin, is altered in 32% of patients with DGC and enriched in 35% of GS subtypes in TCGA cohort, and its germline mutation is a risk factor for hereditary DGC [3, 9]. In addition, *CDH1* hypermethylation is found in approximately 75% of DGC cases; therefore, *CDH1* inactivation is considered to contribute to most DGC cases [10]. *TP53*, a tumor suppressor gene, encodes the transcription factor (TF) coordinating cellular responses to stress elicited by signals, including DNA damage, aberrant growth signaling, and hypoxia. *TP53* is altered in 28% of DGC patients and enriched in 71% of CIN subtypes in the TCGA cohort, demonstrating its impact on genome instability [3]. Although DCKO mouse model has acquired the attribution of two drivers and its histopathological features and gene expression profile are similar to those of human DGC, its genomic profile remains unclear and, therefore, requires further examination for future utilization [8].

In this study, we conducted whole-genome analysis of GC from eight DCKO mice, provided their complete somatic mutation patterns, and compared the results with TCGA PanCancer Atlas human GC. Our results suggest that this DCKO mouse is genetically comparable to human GC and is thus considered an appropriate DGC model mouse for further investigation.

## Materials and methods

### Samples

#### Mouse samples

As previously described [8], the *Atp4b-Cre*^+^; *Trp53*^*loxP/loxP*^; *Cdh1*^*loxP/loxP*^ DCKO mouse was produced by crossing *Cdh1*^*loxP/loxP*^ [11] and *Trp53*^*loxP/loxP*^ [12] mutants (C57BL/6 strain) combined with an *Atp4b-Cre*^+^ transgenic mouse [13] (FVB/NJ strain). PCR-based genotyping confirmed the required genotypes of littermates as previously described [8]. At approximately 12 months of age, the mice were euthanized and their stomachs were resected if they had macroscopically apparent GCs. All GCs found in our mouse cohort were massively invasive tumors, and we collected and stocked representative cancerous tissues as frozen blocks, which included all cancerous layers of the stomach (from the mucosal to the subserosal layers). Duodenal tissues of all mice were also collected and stocked for use as controls for germline sequencing. We then performed histological analysis to confirm that the tissues were genuinely GCs. Genomic DNA was extracted from the frozen archives using a QiaAmp DNA Kit (Qiagen, Venlo, Netherlands) according to the manufacturer’s protocol. Following a DNA quality check via agarose-gel electrophoreses, whole-genome sequencing with paired-end 150-bp lengths was performed using Illumina HiSeq platform (Illumina, Inc., CA, USA). For mouse1 and mouse2, TruSeq Nano DNA Library Prep Kits (Illumina, Inc.) were used to construct sequencing libraries. For all other mice, TruSeq DNA PCR Free Library Prep Kits were used to perform PCR-free whole-genome sequencing. The raw sequence reads are available at the Sequence Read Archive, NCBI (accession no. PRJNA723145).

#### Human samples: TCGA PanCancer Atlas data set

The MuTect2 MAF file of SNV/indels in 437 human gastric cancer patients in TCGA PanCancer Atlas was downloaded from Genomic Data Commons Data Portal [14], whereas the 436 ABSOLUTE [15] copy number segment and ploidy files were downloaded from Genomic Data Commons [16]. The corresponding clinical data for 441 patients, including Lauren classification for 295 patients [3] and molecular subtypes, was downloaded from cBioPortal [17, 18].

### Mapping

Sequence reads were aligned to mouse reference sequence build mm10 using Parabricks [19] 2.5.3 fq2bam, which accelerate BWA-MEM by GPU along with other third party tools. Subsequently, GATK 4.1.4.1 Picard [20] MarkDuplicateSpark was applied for deduplication (PCR-free sample parameters: OPTICAL_DUPLICATE_PIXEL_DISTANCE = 2500; TAGGING_POLICY = OpticalOnly). The deduplicated reads then underwent base quality score recalibration via BaseRecalibrator and ApplyBQSR in Parabricks 2.5.3. Known germline variation of the FVB/NJ strain was downloaded from the Mouse Genomes Project for BaseRecalibrator [21].

### Somatic mutation calling

Mouse single nucleotide variations (SNVs) and indels were extracted by intersecting two callers: (1) Parabricks [19] 2.5.3 mutectcaller, which accelerates GATK Mutect2 [22] by GPU, followed by GATK FilterMutectCalls and (2) Strelka2.9.2 [23]. To improve the reliability of the results, the initially called mutations generated by these two tools were filtered to remove mutant reads found in the corresponding positions of the matched normal sample. Mutations located within chromosomes Y and M were excluded as they are prone to misalignment. The overlapping mutations generated by both callers were acquired for subsequent analyses.

### Mutational burden

The exonic mutational burden was calculated for human and mouse cancers, as well as the whole-genomic mutational burden of the mouse cancers. The mouse and human exon region bed files were downloaded from GENCODE [24] to define exonic mutations. For the mouse mutational burden at the whole-genome scale, the genomic length of the non-N region (except for chromosome Y and M) was calculated using ucsc-facount [25]. Human mutations on chromosome Y were excluded.

### Mutational signature extraction

Both de novo and supervised methods were used to extract mutational signatures from the model mouse. The de novo signatures were determined using Maftools [26]. DeconstructSigs [27] 1.8.0 was applied to extract known signatures from both mouse and human GCs via multiple linear regression models. Human samples with < 30 SNVs were excluded.

For supervised methods, the reference signature set should ideally be estimated via a de novo extraction method to maximize accuracy [28]. Because the mouse signatures extracted using the de novo method were already identified in human GCs, the reference signature set was optimized based on the identified human signatures. First, supervised methods were applied for human GCs using three different reference signature sets: all 30 COSMIC signatures (v2), 11 GC-related signatures (COSMIC signature S1, S2, S5, S13, S15, S17, S18, S20, S21, S26, and S28), and GC-related signatures + S6 [29]. The reconstruction errors were then compared (Supplemental Table 1). We found that these three results were comparable except for MSI samples, where the addition of microsatellite unstable tumor-associated signature (signature 6) drastically decreased the reconstruction error (Supplemental Fig. 1). Thus, we used the combination of human GC-related signatures and signature 6 as the reference for DeconstructSigs in both human and mice analyses.

### Copy number variation detection

#### Absolute copy number

Mouse copy number segments and their absolute values were estimated by cnv_facets [30] v0.15.0. The necessary VCF files of known SNPs of FVB/NJ and C57BL/6NJ strains were downloaded from the Mouse Genomes Project [21] (parameters: -cv 25 400, -g mm10).

Genomic segments with absolute copy numbers ≤ 1 and ≥ 4 were defined as deletion and amplification regions, respectively.

#### Copy number variation fraction, ploidy, and tumor purity

The copy number variation (CNV) fraction was defined as the ratio of the genomic region length bearing CNVs to the total stretch of all CNV regions and non-CNV regions. Mouse and human ploidy and tumor purity information were acquired from cnv_facets [30] and ABSOLUTE [15], respectively. The ploidy and tumor purity from the two methods were considered highly concordant [30].

TCGA GC cohort *TP53* mutation status and its OncoKB annotation were obtained from cBioPortal [17, 18] to evaluate the *TP53* mutation effect on CNV fraction and ploidy. For analysis, samples with oncogenic and likely oncogenic *TP53* annotations were selected as the oncogenic group, whereas other samples were treated as the non-oncogenic group.

#### Chromosomal CNV

If the CNV fraction of a chromosome was ≥ 0.8 and its CNVs were in the same direction (i.e., amplification or deletion, based on absolute copy number), a chromosomal CNV was considered to exist.

#### Focal CNV

Focally amplified or deleted mouse regions were detected by GISTIC 2.0 [31]. The input for GISTIC2.0 included CNV segment data from cnv_facets [30], a MATLAB-formatted reference from MoCaSeq pipeline [32], and pseudomarker data with a probe spacing of ~ 500 bp. Focal CNV thresholds were ≥ 1 for amplification and ≤  −  0.5 for deletion.

The genes targeted by high confidence focal CNV events were calculated by intersecting with cnv_facets absolute copy number results. Focal CNVs with an absolute copy number ≥ 4 or ≤ 1 were retained as amplification or deletion, respectively.

Focal CNV genes from mouse model and human GCs were compared using the gene set that frequently suffered focal CNVs in TCGA GC cohort [3]. Their orthologs (18 amplification and 12 deletion genes) were acquired from NCBI HomoloGene (ftp://ftp.ncbi.nih.gov/pub/HomoloGene/build68/homologene.data). Random CNV profiles were generated (separately for amplification and deletion) 10,000 times by sampling genes (18 and 12 genes for amplification and deletion events, respectively) among all mouse genes. The numbers of CNV events in these generated gene sets were used to determine *p* values. The gene sets harboring the top 5% of CNV events in these 10,000 samplings were considered significant CNV gene sets.

### Mouse structural variation detection

Manta [33] v1.5.0 was used with default parameters to identify somatic structural variations (SVs). Genes targeted by SVs were annotated based on mouse refGene (http://hgdownload.cse.ucsc.edu/goldenpath/mm10/database/refGene.txt.gz). To reduce false positives, we applied the following filters:

- Passed SVs,
- Tumor split read ≥ 1,
- Matched normal split read = 0,
- Matched normal paired read = 0.

### Additional driver gene screening

All SNVs and small indels were annotated using ANNOVAR [34]. Focal CNVs were annotated based on mouse refGene. Altered genes overlapping with known cancer-related genes in OncoKB [35], intOGen [36], and COSMIC cancer gene census [37] were retained, as were genes significantly mutated in TCGA GCs [3]. Additional driver gene candidates were selected according to the following criteria.

SNVs/Indels:

- For genes harboring SNVs/indels, genes with deleterious SNVs or indels, as predicted by either PROVEAN [38] or SIFT [39].
- Genes without known oncogenic mutations were excluded if their functions in the NCBI Gene database [40] were not directly related to GC.

CNVs:

- Genes within the deep deletion (absolute copy number = 0) or high-level amplification (absolute copy number > 99.5 percentile in the distribution of the copy number for each sample).
- The amplification of the tumor suppressor gene, deletion of the oncogene, and genes without any curated oncogenic events were excluded.
- Known GC drivers, genes with extreme CNVs, and recurrently altered genes were preferred, whereas genes were excluded if their functions in the NCBI Gene database [40] were not directly related to GC.

For SNVs and indels located outside the coding regions that hit cis-regulatory elements, recurrent SNVs and indels were extracted and queried in the SCREEN database [41] to find these elements.

### Alteration of the pathway

Mutated pathways were compared between human and mouse GCs by obtaining the oncogenic alterations of curated pathway genes, i.e., SNVs, indels, and CNVs, from TCGA PanCancer Atlas data set [42]. These oncogenic alterations were converted into mouse homologous genes using NCBI HomoloGene. We collected altered mouse genes targeted by SNVs, indels, and focal CNVs detected via GISTIC 2.0 [31] with the same parameters used in TCGA PanCancer Atlas GC cohort [i.e., amplification (+ 2) and deep deletion (− 2)]. The overlap of the mouse CNV genes with curated pathway gene alterations was calculated to acquire mouse oncogenic CNVs. Damaged SNVs and indels in mice were based on PROVEAN [38] or SIFT [39] predictions.

## Results

We applied whole-genome sequencing to paired normal (duodenum) and GC samples of eight DCKO mice. The coverage was as follows: normal = 28–35 × ; tumor = 78–107 × (Supplemental Table 2). Comparisons of SNVs, small indels, CNVs, mutational signatures, altered genes, and pathways in the mouse model with those in four molecular subtypes of human GC (i.e., GS, CIN, EBV, and MSI) from TCGA PanCancer data set are shown below.

### *Trp53*^*−/−*^*Cdh1*^*−/−*^ mice exhibited SNV and indel patterns similar to those in the human GS subtype

We called 22,353 somatic SNVs and 5,568 somatic indels across eight mice (Supplemental Table 3). Samples with PCR libraries (mouse1 and 2) tended to have more SNVs than those with PCR-free libraries (4416–5770 vs. 164–4116 SNVs, respectively), but this trend was not observed for indels (474–698 vs. 18–2807 indels, respectively). The difference in SNVs may be partially derived from PCR error; however, this difference could not be assessed statistically due to low sample numbers.

**Fig. 1:**
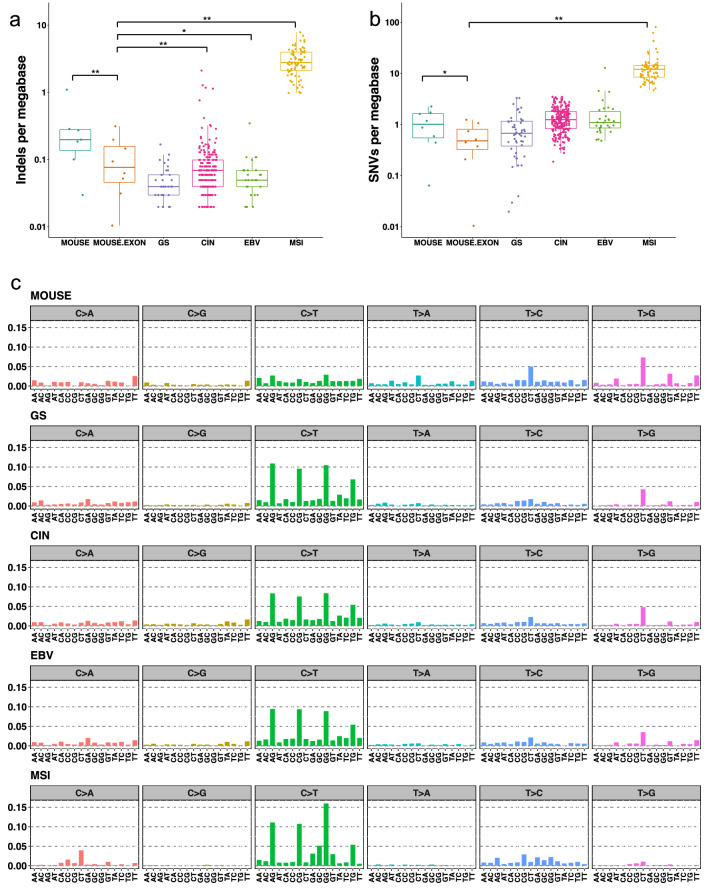
SNV and indel patterns in DCKO mice. (**a** and **b**) Mutational burden in the mouse model at genome and exome scales. Whole-genomic and exonic-scale mutational burdens were compared (Wilcoxon signed-rank test: SNVs, *p* = 0.0078; indels: *p* = 0.023), as was the mouse exonic-scale with human GS, CIN, EBV, and MSI subtypes (Steel multiple comparison Wilcoxon test; SNVs: GS *p* = 0.76, CIN *p* = 0.0032, EBV *p* = 0.018, MSI *p* = 1.5e–5; indels: GS *p* = 0.092, CIN *p* = 0.92 EBV *p* = 0.45, MSI *p* = 1.5e–5). *p < 0.05 and **p < 0.01. **c** Mutational spectra of DCKO mouse and human gastric cancers were determined according to SNVs (C > A, C > G, C > T, T > A, T > C, T > G) and their immediate 3′ and 5′ sequence context (*x-axis* substitution class and sequence context immediately 5′ and 3′ to the mutated base; *y-axis* substitution contribution). Spectra were normalized and created from averages

Estimated tumor purity, 0.15–0.64, was within the human GC purity range [3]. The mouse mutational burden was significantly lower in the exonic (medians of 0.48 SNVs and 0.078 indels per Mb) than in the whole-genomic regions (medians of 1.02 SNVs and 0.19 indels per Mb) (Fig. 1a, b). Such differences are universal across species due to higher mismatch-repair activity/transcription-coupled repair in exonic regions or selection bias [43, 44]. The exonic and genomic mutational burdens of mouse GCs were within the mutational burden range of human GCs but at relatively low levels [45, 46].

Our comparison of mutational burden was limited to the exonic region, because human mutations of GC are generated from whole-exome sequences. The mouse model was found to harbor SNV density that was comparable to that of the human GS subtype, which is a molecular subtype dominated by DGC. In addition, small indel density in mice was similar to that in human GS, CIN, and EBV subtypes [3] (Fig. 1a, b).

We also measured SNVs according to the mutational spectra containing 96 categories of the bases immediately 5’ and 3’ to each single base substitution (Fig. 1c). The mouse model had two characteristic peaks, T > G and T > C substitutions in a CT context, which were also found in human GS, CIN, and EBV subtypes. However, fewer C > T substitutions were found in mouse GCs than were found in human GCs.

### Mutational signatures shared by *Trp53*^*−/−*^*Cdh1*^*−/−*^ mouse and human GCs indicated possible mutual tumorigenic etiologies

**Fig. 2:**
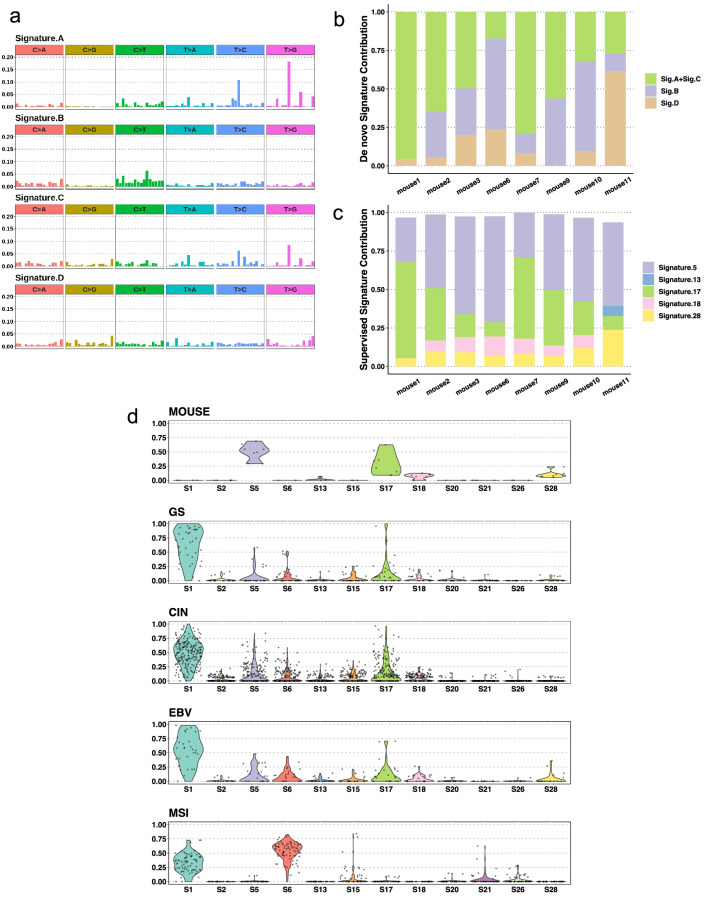
Mutational signatures shared by the model mouse and human gastric cancer. (**a** and **b**) De novo signatures of DCKO mice and their contributions. Signature A and signature C were similar to COSMIC signature 17; signature B and signature D were similar to COSMIC signature 5 and signature 3, respectively (similarities: signature *A* = 0.96, signature *B* = 0.79, signature *C* = 0.77, signature *D* = 0.81). **c** Reconstructed signatures and their contributions extracted by the supervised approach. **d** Distributions of mutational signature contributions in DCKO mice and four human gastric cancer subtypes. Each data point represents the contribution of a signature in one sample

Mutational processes in the mouse model were determined by extracting the signatures that most accurately reconstructed the SNV spectrum of each mouse GC. Although the signatures and their contributions varied among mouse tumors, both de novo and supervised methods detected that signatures 5 and 17 were not only shared by all mice but also contributed to approximately 63–92% of mutations (Fig. 2a–c). The signature distributions from samples with PCR libraries and PCR-free libraries were similar; thus, the contribution of false-positive SNVs derived from PCR error was minor or nonexistent.

Signature 5, which is found in all human cancer types but with unknown etiology, is reported as a clock-like signature in some cancers but not in human GC [47]; therefore, its contribution does not accumulate at a consistent rate in different GC patients. Similarly, its contribution varies in mouse tumor tissues collected from mice of approximately equal ages (~ 12 months). Signature 17 is thought to be caused by Hoogsteen base pair-derived mispairing of 8-oxoG with adenine, which causes T > G substitutions during replication [48, 49]. In conditions involving acid reflux, bile acid along with low pH can induce oxidative stress and cause 8-oxoG [50]. Regarding acid, signature 17 is mainly found in esophagus and gastric adenocarcinomas [45, 46]. Its characteristic peaks of T > C and T > G substitutions were observed in both human and mouse GCs (Fig. 1c). *BRCA*-related signature 3 was also detected using the de novo method. However, we observed few large-scale state transition events, few indel features related to defective homologous recombination-based DNA damage repair (deletions at microhomologies), and a lack of *Brca*-defective alterations in model mice; thus, detection of signature 3 is likely a false-positive discovery (data not shown).

When compared with human GC, mouse tumors had a reproducible but smaller number of mutational signatures except for the MSI subtype (Fig. 2d). Such human–mouse shared signatures indicate possible mutual tumorigenic etiologies, suggesting that mouse represents a simplified human GC model.

### CNV features in DCKO mice were comparable with those in the human CIN subtype

**Fig. 3:**
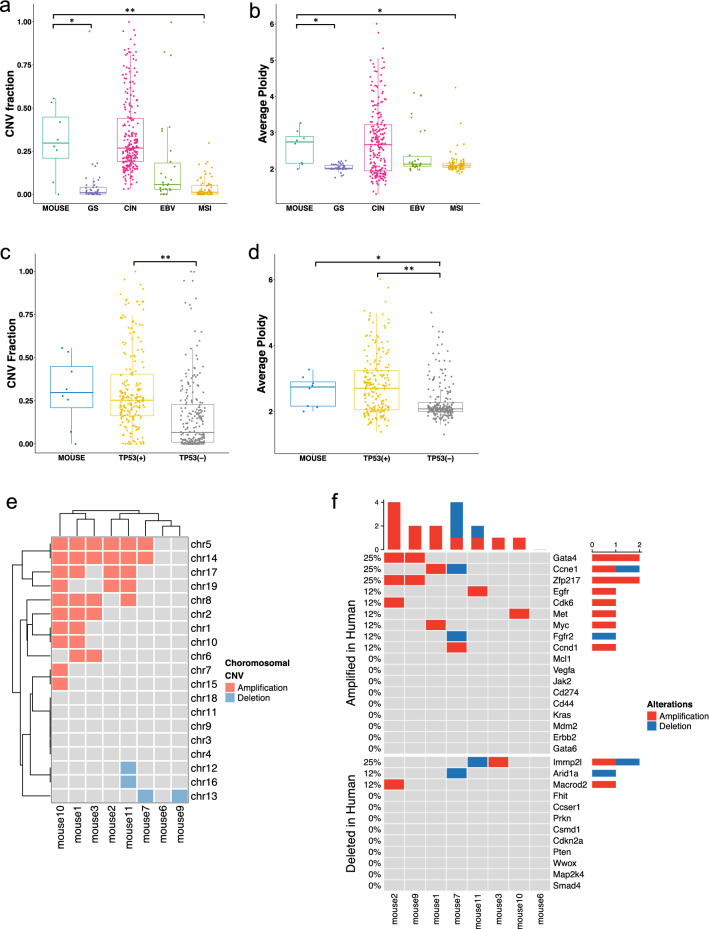
CNV features in DCKO mice were comparable with those in the human CIN subtype. (**a** and **b**) CNV fraction and average ploidy of DCKO mice and four human gastric cancer subtypes (Steel multiple comparison Wilcoxon test. CNV fraction: GS *p* = 0.011, CIN *p* = 1.0, EBV *p* = 0.24, MSI *p* = 0.0066; ploidy: GS *p* = 0.0025, CIN *p* = 1.0, EBV *p* = 0.68, MSI *p* = 0.018). *p < 0.05 and **p < 0.01. **c** and **d** CNV fractions and average ploidy in DCKO mouse and human gastric cancers with or without oncogenic-*TP53*-mutations [Wilcoxon Benjamini–Hochberg test. CNV fraction: mouse-TP53( +), *p* = 0.73; mouse-TP53( −), *p* = 0.058, TP53( +)-TP53( −), *p* = 5.4e-16. Ploidy: mouse-TP53( +), *p* = 0.88; mouse-TP53( −), *p* = 0.033, TP53( +)-TP53( −), *p* = 1.6e-09]. *p < 0.05 and **p < 0.01. **e** Chromosomal CNVs in DCKO mice. Mouse chromosomal CNVs were hierarchically clustered with amplification set as 1, deletion as − 1, and other as 0 (Euclidean distance, average method). **f** Focal CNV status of mouse orthologs of human genes with frequent focal amplifications or deletions in gastric cancers (compared with random events; amplification: *p* = 9e–4; deletion: *p* = 0.62)

Median CNV fraction and ploidy were 0.30 and 2.75 in mouse tumors, respectively, which are similar to those in human CIN and EBV subtypes but significantly higher than those in GS and MSI subtypes (Fig. 3a, b). Human CIN and GS subtypes are classified by their high and low somatic CNV status, respectively [3]. These features indicate that the mouse model genome has undergone frequently broad-CNV events leading to chromosomal instability. Although CIN is not diffuse-type dominant, approximately 40% of DGCs in TCGA cohort were classified under CIN. By comparing CNV fraction and ploidy between mice and human GCs with or without oncogenic-*TP53*-mutations, the mutation significantly increased ploidy in human GCs and were no significant differences were seen between the mice and the oncogenic-*TP53*-mutant human GCs (Fig. 3c, d). Therefore, *Trp53* knockout may be responsible for chromosomal instability in DCKO mice.

Considering chromosomal CNVs, the model mice had more amplifications than deletions (Fig. 3e). Chromosomal amplification most frequently occurred in chromosomes 5 and 14, which were largely coamplified (6/8 cases), whereas chromosomal deletions were rare and most often occurred in chromosome 13. Such preferential amplification or deletion of specific chromosomes is also observed in human GCs and other cancer model mice (although such events occur in different chromosomes), implying that the process contributes to tumorigenesis [3, 51].

Considering mouse orthologs of the most common CNV genes in human GCs, focal CNVs were found to target some of these genes and most were found in amplified genes, including *Gata4*, *Ccne1*, *Zfp217*, *Egfr*, *Cdk6*, *Met*, *Myc*, and *Ccnd1* (Fig. 3f). Base on a statistical model of randomly generated CNV events, these commonly amplified genes in human GCs also had significantly frequent focal amplifications in the mouse model.

### Mouse orthologs of human common fragile site (CFS) genes were frequently subjected to SV breakpoints

According to the Circos plots, B-allele frequencies, and copy number status, we identified four mice that might have chromothripsis (Supplemental Figs. 2, 3) [52]. From the aspect of SV breakpoints, 27 genes were recurrently targeted (Supplemental Fig. 4a, Supplemental Table 4). *Wwox*, *Fhit*, and *Macrod2*, the human orthologs of which were the most frequent CFSs targeted by SV in GCs, were altered in 5, 2, and 2 mice, respectively (Supplemental Fig. 4b) [53]. Particularly, *Wwox* and *Fhit* were confirmed as CFSs in mice [54, 55]. SV breakpoints were also identified in seven other orthologs of human CFS genes: *Immp2l*, *Negr1*, *Naaladl2*, *Ccser1*, *Prkn*, *Lsamp*, and *Gpc6*. Seven of eight mice exhibited at least one SV in the human CFS gene ortholog; similar to human cancers, deletion and duplication were the dominant alterations in these genes [53]. Previously, genome-wide analysis of mouse CFSs was completed only in neural stem/progenitor cells [56]. Thus, this is the first genome-wide report that describes mouse orthologs of human CFS genes frequently subjected to SV breakpoints in mouse cancer.

### Additional driver screening discovered well-known drivers and lineage-specific TFs

**Fig. 4:**
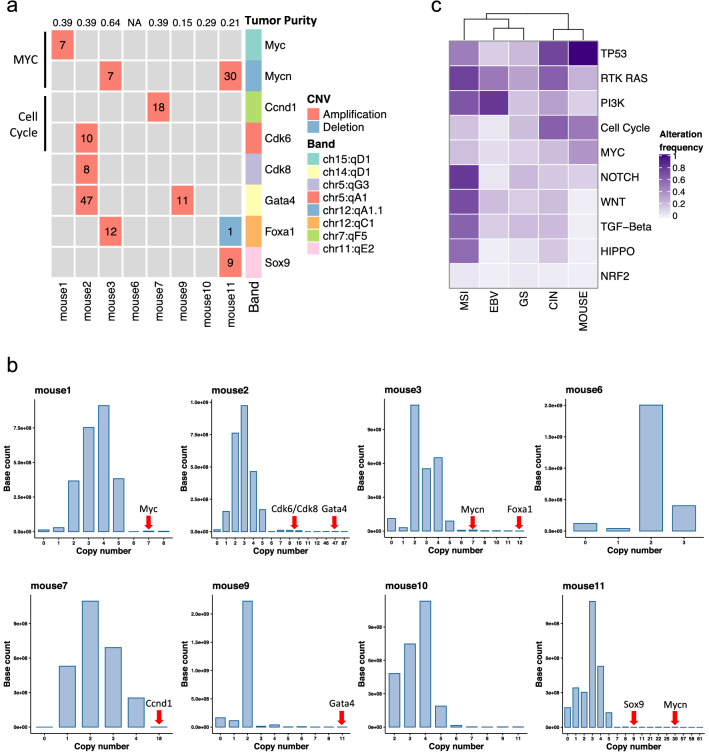
Additional drivers and an overview of the mutant pathway. (**a**) Putative additional drivers identified in (**b**). Numbers in grids indicate the abnormal absolute copy number of each gene. **b** Histograms showing the distribution of base-level absolute copy number frequency for each mouse. Possible additional drivers were identified based on the distribution and previous knowledge of the gene candidates (see Materials and Methods). **c** Mutation pattern of the DCKO mouse and human gastric cancers. Pathway alteration frequency indicates the percentage of samples with one or more genes altered within a specific pathway (hierarchical clustering using Euclidean distance)

Cancer-related genes were screened to identify additional drivers based on cancer-related gene databases and functions in the cancer context. Oncogenic SNVs/indels were not identified in the model mice (Supplemental Table 5) nor was the candidate cis-regulatory element hit by recurrent SNVs/indels. CNV events were dominant in altered cancer-related genes, indicating that CNVs play a crucial role in GC formation in the model mouse. Potential additional drivers were identified by high-level amplifications: *Myc*, *Cdk6*, *Cdk8*, *Gata4*, *Mycn*, *Foxa1*, *Ccnd1*, and *Sox9* in six of eight mice (Fig. 4a, b; Supplemental Fig. 5a, Supplemental Table 6).

Within the recurrently altered genes, the focal amplification of *Mycn* and *Gata4*, targeted by very high-amplitude amplifications, was notable. *GATA4*, a TF involved in the development and differentiation of the gastrointestinal tract that may function in carcinogenesis [57, 58], was recurrently amplified in human GC [29 cases (6.62%) in TCGA cohort]. *MYCN*, a *MYC* paralog, is frequently amplified in human neuroblastoma and was amplified in three human GCs in TCGA cohort [59]. *Myc* and *Mycn* amplifications were mutually exclusive in model mice and human GCs. Other gastrointestinal TFs, including *Foxa1* and *Sox9*, were also targeted by focal amplifications. *Foxa1* establishes competence in the foregut endoderm and plays roles in mammary and prostate cancer [60]. Although only three human GCs in TCGA cohort exhibited *FOXA1* amplification (Supplemental Fig. 5b), two were focal and no other genes related to GC were found in the regions; thus, *FOXA1* amplification may be a GC driver event. The focal copy number status of TFs related to gastric development was further investigated (Supplemental Table 7) [57, 58, 61]. Although other well-known gastrointestinal TFs were not altered, lineage-specific TFs are likely involved in GC formation and the model mouse captured this feature [58, 62, 63].

Pathway alteration analysis showed that, other than the 100% altered TP53 pathway, the cell cycle and MYC pathways were the oncogenic pathways primarily altered in model mice, with alteration frequencies of 0.5 and 0.38, respectively (Fig. 4c). CNVs contributed to nearly all of these alterations. The hierarchical clustering demonstrated that pathway alteration was similar in mice and human CIN subtype, consistent with CNV as the predominant characteristic of the mice.

## Discussion

In summary, we have analyzed the genomic features and underlying etiologies of the *Cdh1/Trp53* DCKO model mouse, demonstrated its similarities with human GCs, and provided information to aid future research using this model.

In the mouse model, signatures 5 and 17 were especially reproducible and both were highly prevalent in human GC; therefore, the mouse model could potentially be used to study the underlying etiology of mutational processes, e.g., that of signature 17 as the characteristic signature in gastric and esophagus adenocarcinoma. The mechanism underlying signature 17, which may involve acid-induced oxidative stress, remains to be experimentally verified due to the lack of an experimental model, which may be rectified by the DCKO mouse model [48]. Signatures 1 and 5 (“clock-like signatures”) manifest in all cancer types and most human cancer samples; however, DCKO mice harbored only signature 5, as observed in other cancer mouse models [51, 64, 65]. The absence of signature 1 explains why fewer C > T substitutions were found in mice than in human GCs (Fig. 1c). There are two possible explanations for the absence of signature 1. First, although spontaneous 5-methylcytosine deamination caused by aging is a possible etiology of signature 1, recent research indicates that chronic inflammation of *Helicobacter pylori* can induce methylation at CpG islands, which may be another leading source of C > T substitutions at NpCpG, i.e., the typical feature of signature 1 [66]. Because the DCKO mice did not have *H. pylori* infections, aging could be the only cause. Second, spontaneous 5-methylcytosine deamination that increases with cell mitosis requires a long time to accumulate [47, 67, 68]; the experimental period (~ 12 months) was likely too short for the model mice to acquire a comparable level of signature 1 to that found in human patients.

When classifying all mouse and human samples by their signatures, they were divided into four clusters: signature 17, 1, 5, and 6 prevalent clusters (Supplemental Fig. 6). Mouse samples were clustered in the signature 5 (c2) and 17 (c4) clusters, whereas the signature 6 cluster (c1) was dominated by the human MSI subtype that showed a distinct genomic character. The c2, c3, and c4 clusters did not differ significantly in terms of molecular subtypes, Lauren classification, tumor stage, and age. Although no evidence existed for relationships between signatures and other features, surprisingly, mouse samples were gathered into two human clusters instead of forming an independent cluster. This result suggests that this mouse model mimics the heterogenicity of human GS, CIN, and EBV GCs in terms of their etiology for the mutation process.

In DCKO mice, the tumor mutational burden was low and comparable with the human GS subtype, suggesting that lower levels of neoantigens could have led to immunologically cold tumors, as observed in the GS subtype [69]. For driver genes other than *Trp53* and *Cdh1*, strong oncogenic mutations caused by SNVs or indels were not observed; oncogenic alterations in the mice were dominated by CNVs, while pathway alterations were most similar to the CIN subtype. Chromosomal instability in the mouse model might be induced by *Trp53* deletion, which can cause centrosome amplification leading to chromosome mis-segregation and chromosomal instability [70, 71]. The amplification preference CNV pattern may be caused by positive selection or abnormal chromosome segregation/nondisjunction during mitosis. We observed multiple cancer-related genes on broad-amplified chromosomes, e.g., chromosomes 5 and 14 (data not shown), despite only some cancer-related genes on these two chromosomes having focal CNVs, such as *Gata4*, *Cdk6*, and *Cdk8*. These co-occurring amplified cancer-related genes might act as drivers during cancer transformation [72]. Besides, E-cadherin loss leads to the loss of cell–cell adhesion and anchorage-independence, i.e., essential steps in the development of tumors with diffuse-type histology. Furthermore, motile nontumor cells were reported in a mouse model with *Cdh1* conditionally knocked out in parietal cells, suggesting that anoikis-resistance in DCKO mice may be induced by *Cdh1* knockout (although the underlying mechanism is unknown) [73].

Although two crucial driver genes, *Cdh1* and *Trp53,* were knocked out in DCKO mice, at least 6 months were required to develop GCs; hence, additional drivers are necessary for tumorigenesis [8]. Gastrointestinal TFs (*Gata4*, *Sox9*, and *Foxa1*) were targeted by typical focal amplifications in 50% of mice (four of eight) and 15% of humans (Supplemental Table 7). CNVs or abnormal expression in lineage-specific TFs are known to be important for the formation of GCs, although their function seems somewhat paradoxical [58, 62]. In TCGA GC cohort, *GATA4* alterations were dominated by amplifications, indicating oncogenicity; likewise, all *Gata4* alterations in mice were amplifications [3, 58]. Contrastingly, *SOX9* alteration effects remain to be clarified; however, recent research shows the redundant role of *Sox2* and *Sox9* in GC development, providing some explanation for the conflicting result [3, 61]. In TCGA cohort, lineage-specific TF alterations were found in all GC subtypes with no distinct enrichment of specific morphological or molecular classification; this might, therefore, be a general feature of GC. In the mouse model, alterations in some lineage-specific TFs (such as *Gata6*) found in human GCs could not be identified; analyzing more mice, however, might lead to the discovery of other lineage-specific TFs.

In this study, we focused on advanced GCs. However, it would also be interesting to investigate the genomic profiles of intramucosal tumors to identify early driver events in the future.

In conclusion, the DCKO mouse not only shared morphology with human DGCs but also captured the essential aspects of human GCs, including mutational process, abnormal chromosomal profile, and driver genes. Therefore, it can be considered a suitable DGC model mouse for future research.

## Supplementary Information

Below is the link to the electronic supplementary material.

Supplementary file1 (PDF 10217 KB)

Supplementary file2 (XLS 128 KB)

Supplementary file3 (XLS 604 KB)

Supplementary file4 (XLS 56 KB)

Supplementary file5 (XLS 496 KB)
