# Supplementary material for: Genomic landscape of a mouse model of diffuse-type gastric adenocarcinoma
Source: Gastric Cancer. 2021 Aug 13;25(1):83–95. doi: 10.1007/s10120-021-01226-0 (PMC8732846; doi:10.1007/s10120-021-01226-0)
Supplement: Supplementary file 1 — Supplementary file1 (PDF 10217 KB) [file 10120_2021_1226_MOESM1_ESM.pdf]

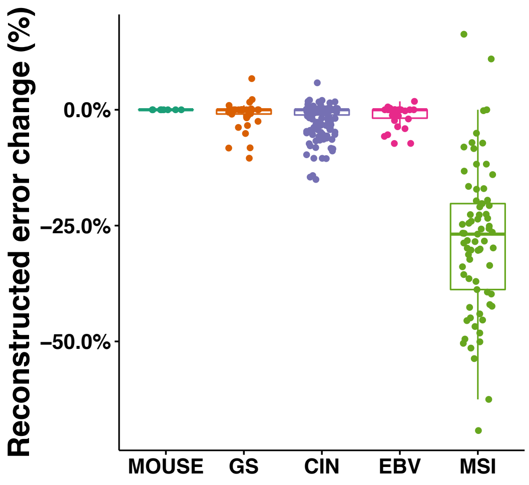

Supplemental Fig. 1. Change in reconstructed errors according to different reference signature sets. The vertical axis represents the decreased ratio of reconstructed errors by gastric cancer-related signatures and signature 6 (error_s12) compared with that by gastric cancer-related signatures only (error_s11). The reconstructed error for each sample is shown in Supplemental Table 1.

Supplemental Table 2. Sequencing statistics

Sample*	Mapped reads	Total reads	Mapping rate (%)	Average depth (×)	Coverage at least 1× (%)	Coverage at least 5× (%) **
mouse1N†	686,424,007	690,189,248	99.5	32.4	97.9	96.9
mouse1T†	1,760,067,996	1,768,951,232	99.5	77.8	98.4	97.5
mouse2N†	572,274,358	575,959,334	99.4	27.8	96.1	95.6
mouse2T†	1,707,299,805	1,727,069,406	98.9	77.6	96.2	95.9
mouse3N	694,786,902	878,539,574	90.5	34.6	95.1	94.2
mouse3T	2,096,591,729	2,679,937,228	91.4	105.0	95.4	94.7
mouse6N	673,148,693	866,240,228	91.8	34.2	94.9	94.0
mouse6T	1,994,377,732	2,565,590,330	91.1	99.3	95.3	94.6
mouse7N	680,350,881	902,993,898	91.5	34.8	95.0	94.1
mouse7T	1,959,871,069	2,473,695,076	91.6	99.3	95.2	94.6
mouse9N	678,122,173	877,817,678	91.9	34.6	95.0	94.1
mouse9T	2,058,843,749	2,628,230,392	91.1	102.8	95.5	94.7
mouse10N	675,436,008	883,001,728	91.2	34.1	95.0	94.1
mouse10T	2,027,119,891	2,602,551,374	91.6	102.2	95.3	94.7
mouse11N	666,863,094	855,346,382	91.3	33.3	94.9	94.0
mosue11T	2,114,498,609	2,738,673,484	91.8	107.0	95.3	94.7

*: N: normal tissue; T: tumor tissue.

**: For mouse1 and mouse2: coverage at least 4×.

†: Sequenced by PCR libraries; the other samples were sequenced by PCR-free libraries.

Supplemental Table 3. Number of SNVs and indels detected in eight mouse tumors

Mouse1 and mouse2 were sequenced by PCR libraries; the other samples were sequenced by PCR-free libraries.

Sample	SNV	Indel
mouse1	4,416	474
mouse2	5,770	698
mouse3	4,116	2,807
mouse6	164	18
mouse7	3017	257
mouse9	1133	76
mouse10	1511	506
mouse11	2,226	732
Total	22,353	5,568

mouse1

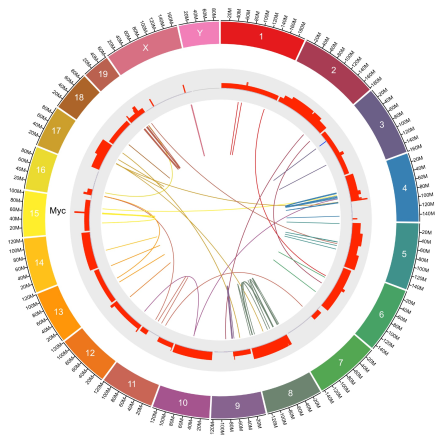

mouse2

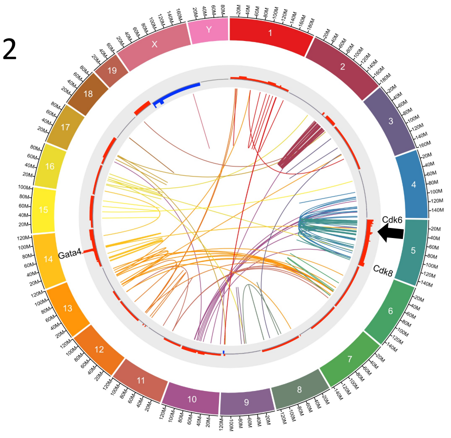

mouse3

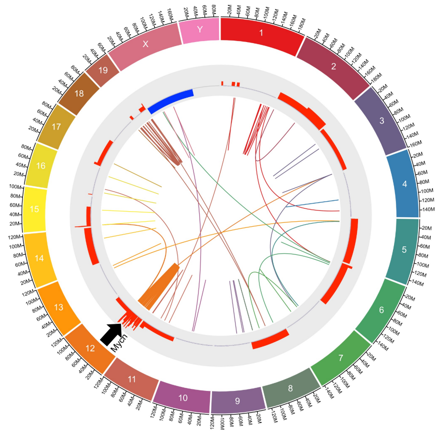

mouse6

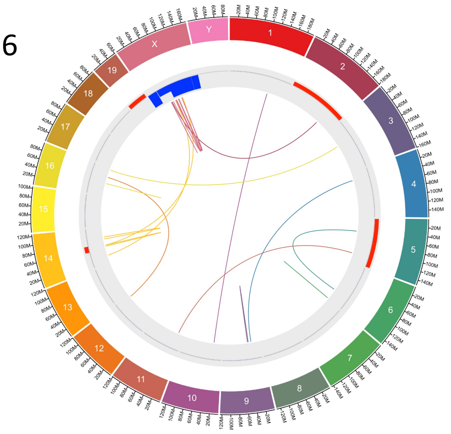

mouse7

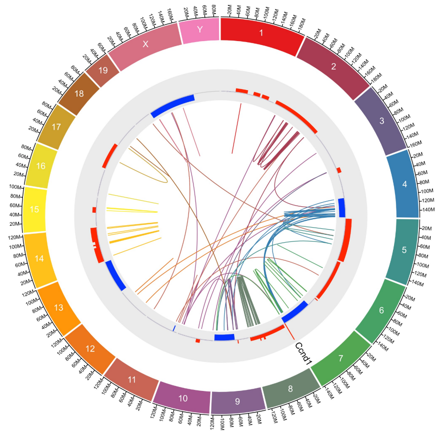

mouse9

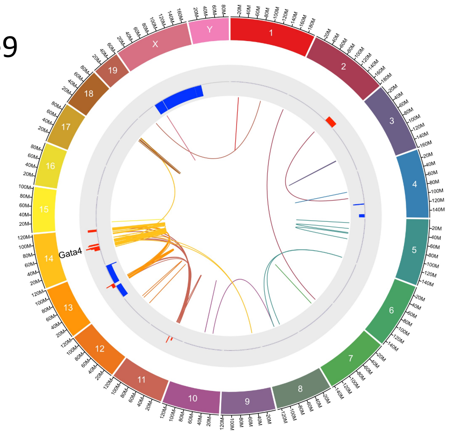

mouse10

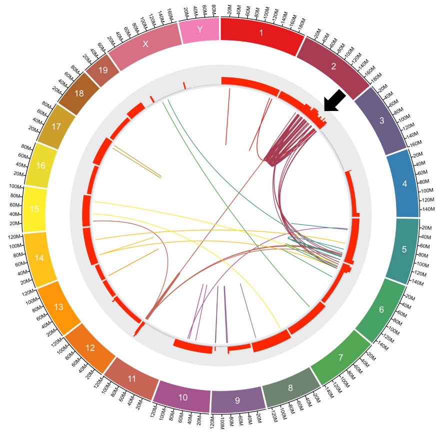

mouse11

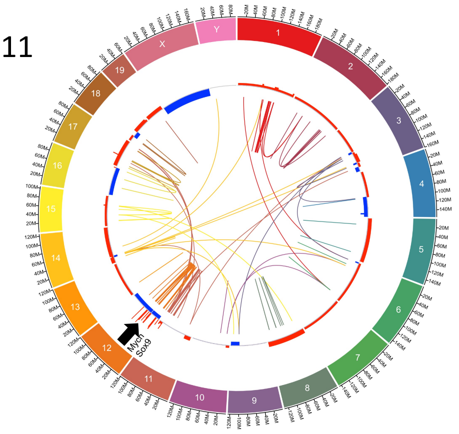

Supplemental Fig. 2. Circos plot for eight mouse tumors. Black arrows indicate potential chromothripsis. Outer ring: chromosome loci. Middle ring: absolute copy number, red: amplification; blue: deletion. Inner ring: structural variation, color indicates the chromosome at which the first broken end of SV starts.

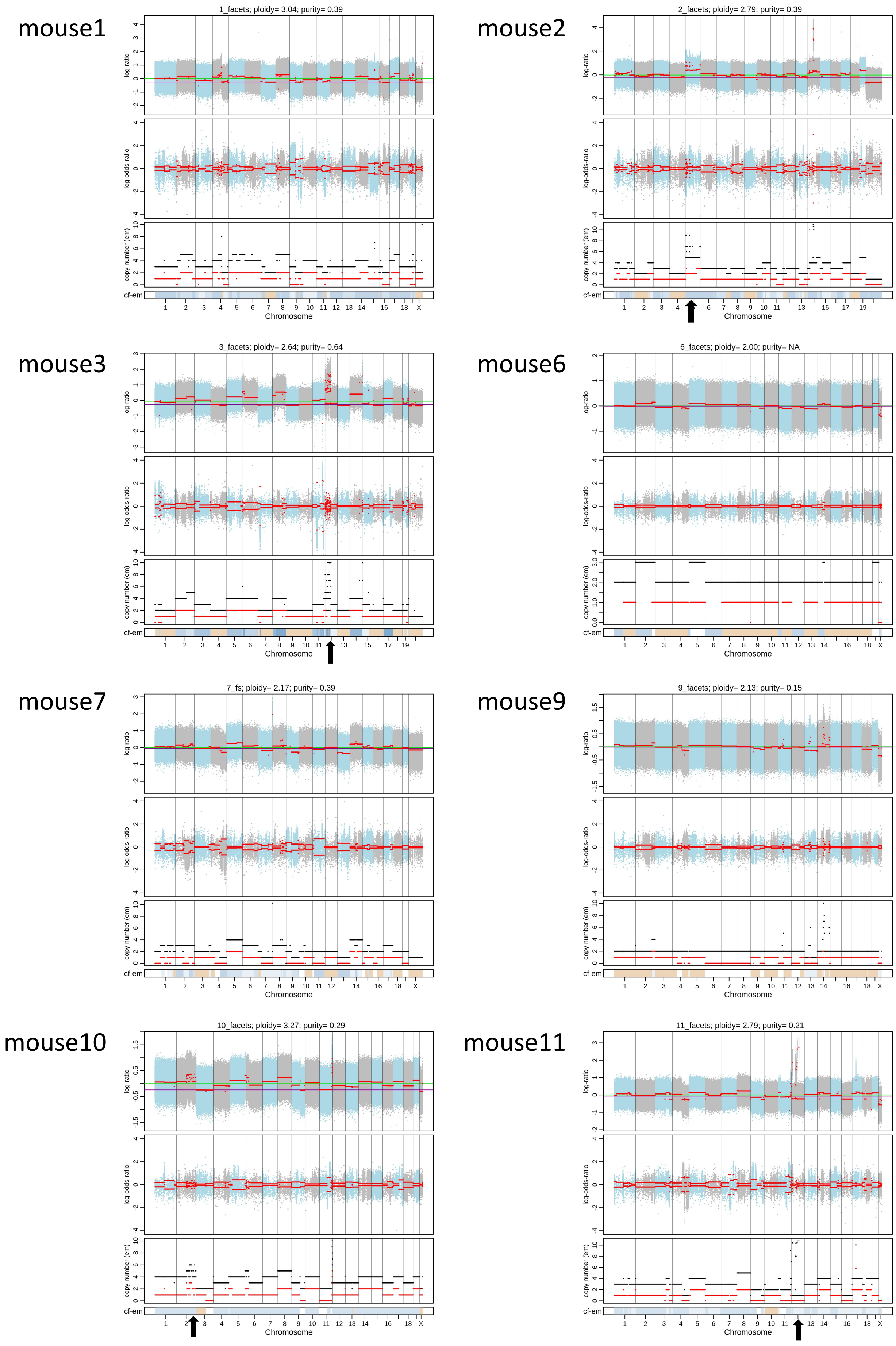
mouse1
1_facets; ploidy= 3.04; purity= 0.39
mouse2
2_facets; ploidy= 2.79; purity= 0.39
mouse3
3_facets; ploidy= 2.64; purity= 0.64
mouse6
6_facets; ploidy= 2.00; purity= NA
mouse7
7_fs; ploidy= 2.17; purity= 0.39
mouse9
9_facets; ploidy= 2.13; purity= 0.15
mouse10
10_facets; ploidy= 3.27; purity= 0.29
mouse11
11_facets; ploidy= 2.79; purity= 0.21
log-ratio
log-odds-ratio
copy number (em)
cf-em
Chromosome

Supplemental Fig. 3. B-allele frequencies and copy number status of eight mouse tumors. Black arrows indicate potential chromothripsis.

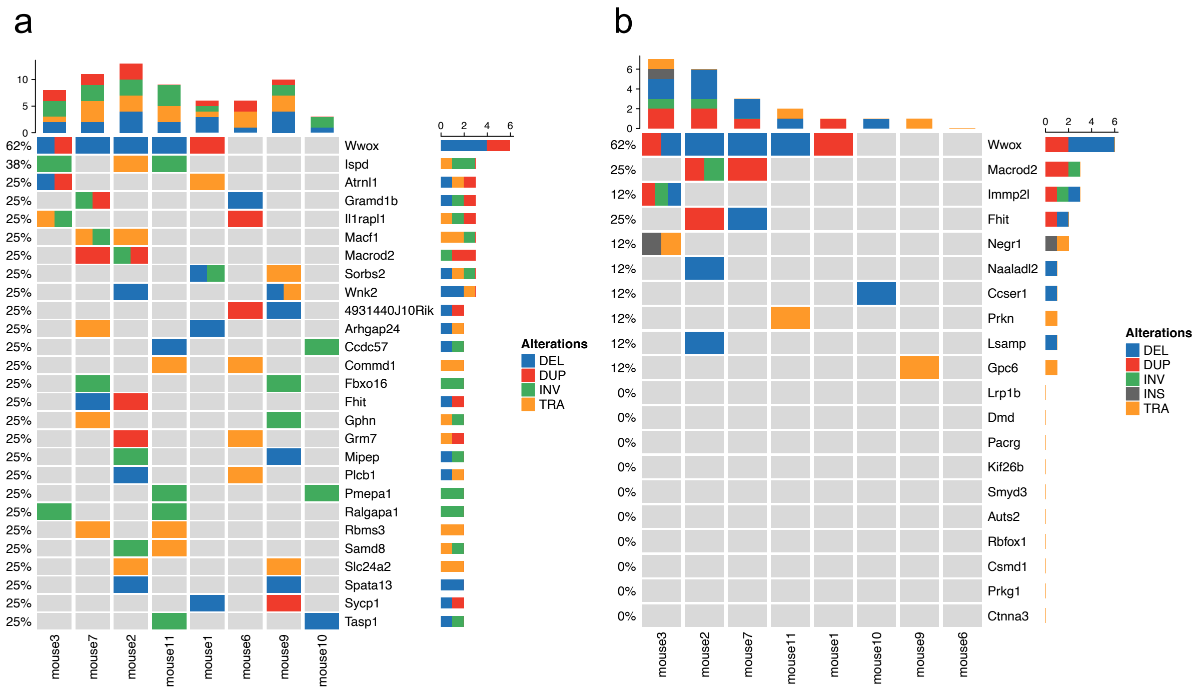

Supplemental Fig. 4. SVs in DCKO mice. (a) Genes targeted by recurrent SVs. **(b)** SV status of mouse orthologs of human CFS genes.

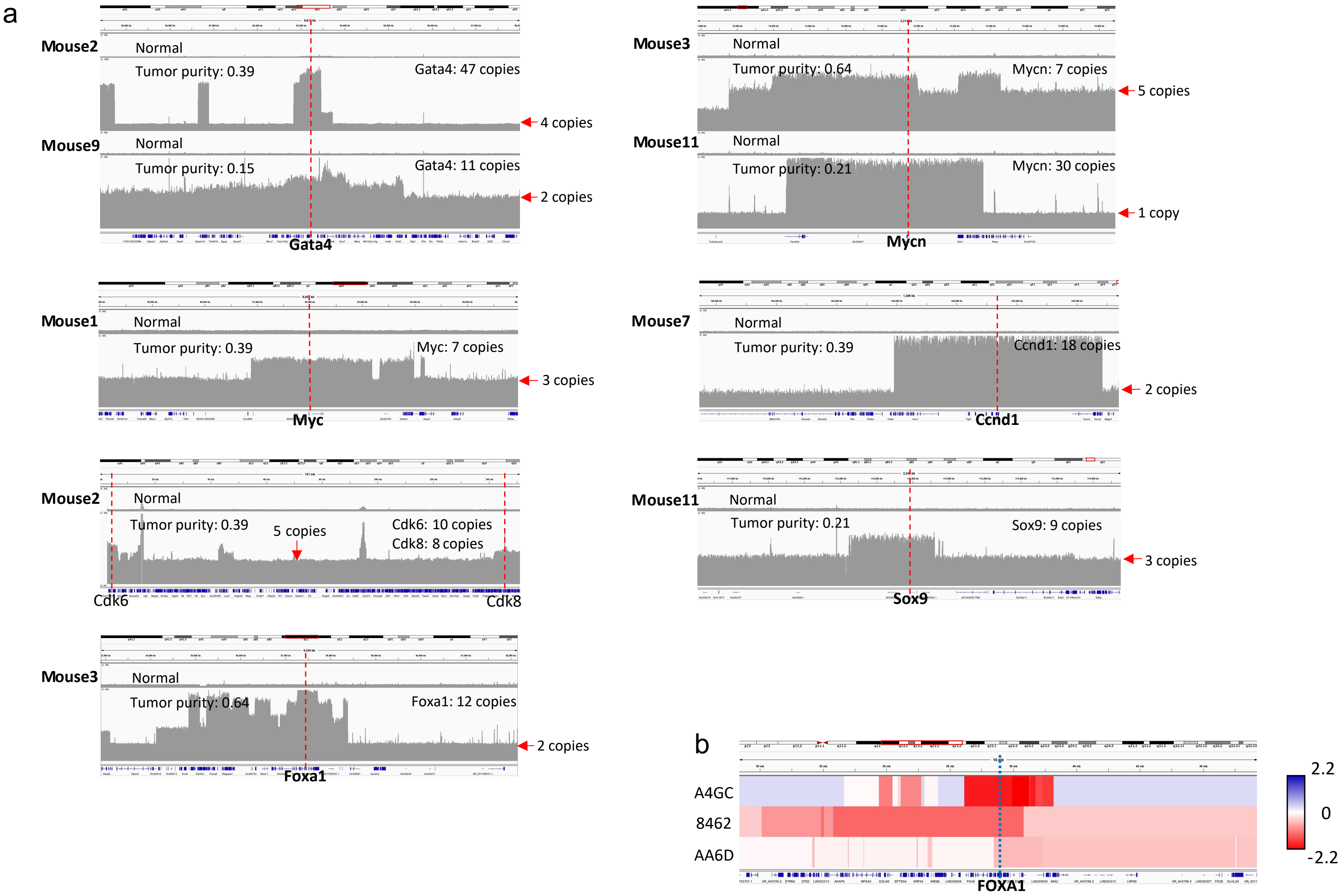
a
Mouse2
Normal
Tumor purity: 0.39
Gata4: 47 copies
4 copies
Mouse9
Normal
Tumor purity: 0.15
Gata4: 11 copies
2 copies
Gata4
Mouse3
Normal
Tumor purity: 0.64
Mycn: 7 copies
5 copies
Mouse11
Normal
Tumor purity: 0.21
Mycn: 30 copies
1 copy
Mycn
Mouse1
Normal
Tumor purity: 0.39
Myc: 7 copies
3 copies
Myc
Mouse7
Normal
Tumor purity: 0.39
Ccnd1: 18 copies
2 copies
Ccnd1
Mouse2
Normal
Tumor purity: 0.39
5 copies
Cdk6: 10 copies
Cdk8: 8 copies
Cdk6
Cdk8
Mouse11
Normal
Tumor purity: 0.21
Sox9: 9 copies
3 copies
Sox9
Mouse3
Normal
Tumor purity: 0.64
Foxa1: 12 copies
2 copies
Foxa1
b
A4GC
8462
AA6D
FOXA1
2.2
0
-2.2

Supplemental Fig. 5. Focal amplifications in putative additional drivers. (a) Focal amplifications and estimated absolute copy number in each putative additional driver. **(b)** Focal amplification of *FOXA1* in three TCGA gastric cancers (IDs: TCGA-HU-A4GC, TCGA-IN-8462, and TCGA-VQ-AA6D).

Supplemental Table 7. Focal CNV status of gastrointestinal TFs. Numbers in the table indicate the absolute copy numbers of genes targeted by focal CNV events.

Sample	Gata4	Gata6	Klf5	Sox2	Sox9	Foxa1
	(14qD1)	(18qA1)	(14qE2.2)	(3qA3)	(11qE2)	(12qC1)
mouse1	-	-	-	-	-	-
mouse2	47	-	-	-	-	-
mouse3	-	-	-	-	-	12
mouse6	-	-	-	-	-	-
mouse7	-	-	-	-	-	-
mouse9	11	-	-	-	-	-
mouse10	-	-	-	-	-	-
mouse11	-	-	-	-	9	1

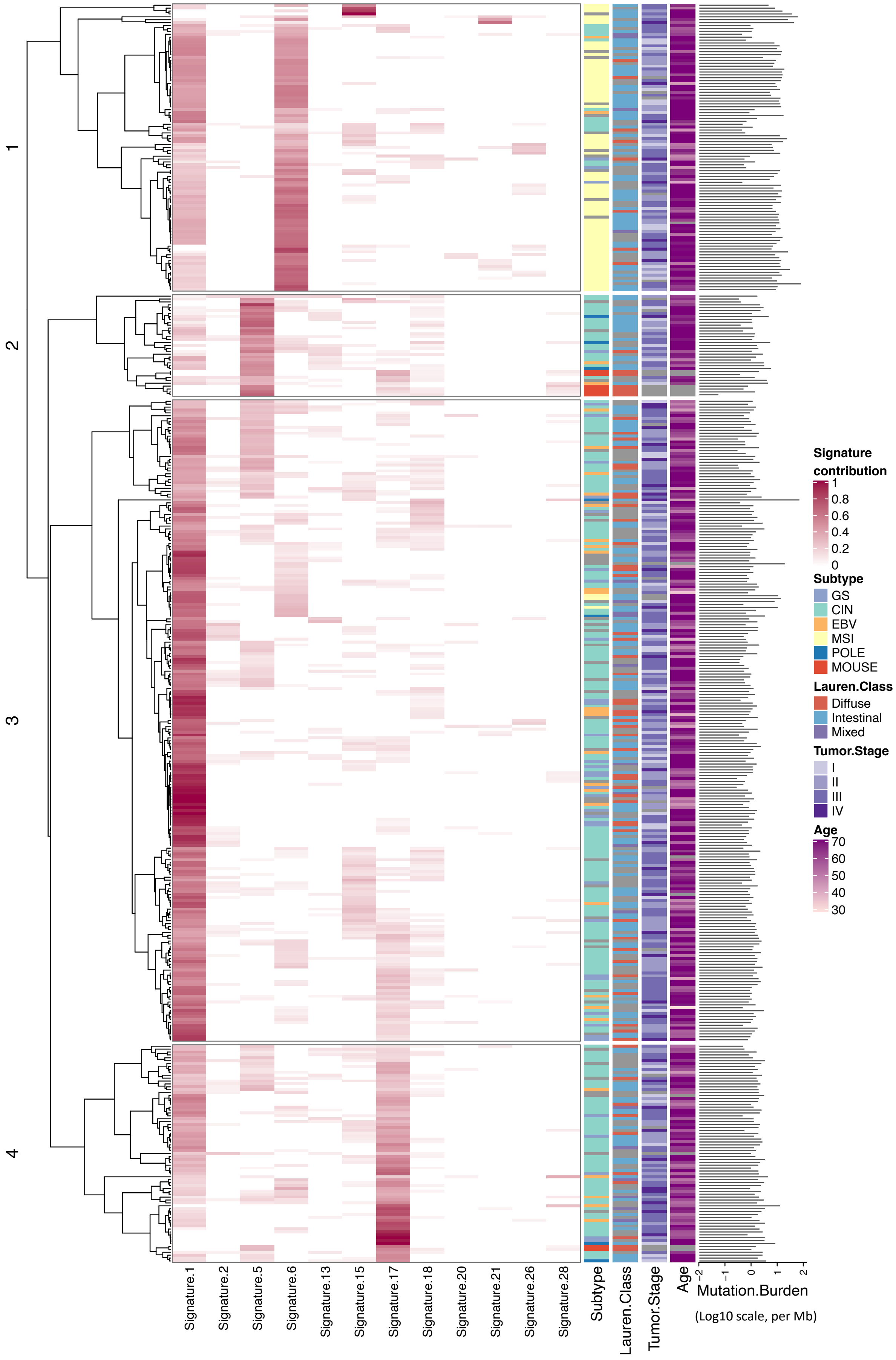
1
2
3
4
Signature.1
Signature.2
Signature.5
Signature.6
Signature.13
Signature.15
Signature.17
Signature.18
Signature.20
Signature.21
Signature.26
Signature.28
Subtype
Lauren.Class
Tumor.Stage
Age
-2 -1 0 1 2
Mutation.Burden
(Log10 scale, per Mb)
Signature contribution
1
0.8
0.6
0.4
0.2
0
Subtype
GS
CIN
EBV
MSI
POLE
MOUSE
Lauren.Class
Diffuse
Intestinal
Mixed
Tumor.Stage
I
II
III
IV
Age
70
60
50
40
30

Supplemental Fig. 6. Signature-based hierarchical clustering of mouse and human gastric cancers

All cases were classified into four clusters: signature 6 cluster (c1), signature 5 cluster (c2), signature 1 cluster (c3), and signature 17 cluster (c4). There were no significant differences in clinical features among c2, c3, and c4 (chi-square test, subtype: $p = 0.24$; Lauren class: $p = 0.70$; tumor stage: $p = 0.58$; Kruskal–Wallis rank sum test, age: $p = 0.46$). NA: gray.
